# Assessment of the Composition Effect of a Bio-Cementation Solution on the Efficiency of Microbially Induced Calcite Precipitation Processes in Loose Sandy Soil

**DOI:** 10.3390/ma16175767

**Published:** 2023-08-23

**Authors:** Joanna Fronczyk, Nadella Marchelina, Adam Pyzik, Małgorzata Franus

**Affiliations:** 1 Institute of Civil Engineering, Warsaw University of Life Sciences—SGGW, 166 Nowoursynowska Str., 02-787 Warsaw, Poland; 2 Faculty of Civil Engineering and Architecture, Lublin University of Technology, 40 Nadbystrzycka Str., 20-618 Lublin, Poland; d570@pollub.pl (N.M.); a.pyzik@pollub.pl (A.P.); m.franus@pollub.pl (M.F.)

**Keywords:** microbially induced calcite precipitation, soil improvement, calcite, bio-stabilization, loose sandy soil

## Abstract

Soil properties are the most important factors determining the safety of civil engineering structures. One of the soil improvement methods studied, mainly under laboratory conditions, is the use of microbially induced calcite precipitation (MICP). Many factors influencing the successful application of the MICP method can be distinguished; however, one of the most important factors is the composition of the bio-cementation solution. This study aimed to propose an optimal combination of a bio-cementation solution based on carbonate precipitation, crystal types, and the comprehensive strength of fine sand after treatment. A series of laboratory tests were conducted with the urease-producing environmental strain of bacteria *B. subtilis*, using various combinations of cementation solutions containing precipitation precursors (H_2_NCONH_2_, C_6_H_10_CaO_6_, CaCl_2_, MgCl_2_). To decrease the environmental impact and increase the efficiency of MICP processed, the addition of calcium lactate (CaL) and Mg ions was evaluated. This study was conducted in Petri dishes, assuming a 14-day soil treatment period. The content of water-soluble carbonate precipitates and their mineralogical characterization, as well as their mechanical properties, were determined using a pocket penetrometer test. The studies revealed that a higher concentration of CaL and Mg in the cementation solution led to the formation of a higher amount of precipitates during the cementation process. However, the crystal forms were not limited to stable forms, such as calcite, aragonite, (Ca, Mg)-calcite, and dolomite, but also included water-soluble components such as nitrocalcite, chloro-magnesite, and nitromagnesite. The presence of bacteria allowed for the increasing of the carbonate content by values ranging from 15% to 42%. The highest comprehensive strength was achieved for the bio-cementation solution containing urea (0.25 M), CaL (0.1 M), and an Mg/Ca molar ratio of 0.4. In the end, this research helped to achieve higher amounts of precipitates with the optimum combination of bio-cementation solutions for the soil improvement process. However, the numerical analysis of the precipitation processes and the methods reducing the environmental impact of the technology should be further investigated.

## 1. Introduction

Microbially induced carbonate precipitation (MICP) refers to a bio-mineralization process in which microorganisms in rock masses or soil matrix produce calcium carbonate mineral crystals [1]. The MICP process occurs naturally in various situations and can be induced artificially under appropriate environmental and nutritional conditions in order to benefit from the good cementing qualities produced throughout the process [2]. MICP technology has been developed for various types of soil, such as sand [3], expansive soils [4], loess [5], and silt [6], and has been successfully applied in many engineering areas related to stability and erosion prevention, such as wind erosion resistance [7], mitigation of beach erosion [8], and rainfall erosion resistance [9]. The MICP technique is classified into six types depending on the pathway used, such as ureolysis, photosynthesis, denitrification, ammonification, sulfate reduction, and organic compound oxidation by bacterial metabolism [10], among which only denitrification and sulfate reduction occur under anaerobic conditions and are difficult to apply under unsaturated conditions in soil. However, in application to soil stabilization, urea hydrolysis is mostly used due to the shorter period required to obtain a significant amount of precipitation and the higher efficiency of the process [11]. This process requires non-pathogenic bacteria strains with high urease production efficiency, such as *B. pasteurii*, *S. pasteurii*, *Sporolactobacillus*, *Clostridium*, and *Desulfotomaculum* [12]. The enzyme released by bacteria into the soil matrix (urease) enhances hydrolysis, resulting in the formation of ammonium (NH_3_), which is then decomposed into ammonium ions (NH_4_^+^) [13], and carbonate ions (CO_3_^2−^) [14]. The reactions taking place can be described by the following equations (from Equations (1)–(3)):

(NH_2_)_2_CO + (H_2_O) → (2NH_3_) + (H_2_CO_3_), (1)

(2NH_3_) + (2H_2_O) ↔ (2NH^4+^) + (2OH^−^) (2)

H_2_CO_3_ ↔ (HCO_3_^−^) + H^+^ ↔ CO_3_^2−^ + 2H^+^ (3)

Furthermore, in the presence of calcium and magnesium sources, the alkaline environment triggers the precipitation of calcium carbonate (CaCO_3_) [15,16], magnesium calcite, or dolomite MgCa(CO_3_)_2_ [17].

Ca^2+^ + CO_3_^2−^ → CaCO_3_ (solid) (4)

Ca^2+^ + Mg^2+^ + 2CO_3_^2−^ → MgCa(CO_3_)_2_ (solid) (5)

Another pathway to produce carbonate precipitation is by using bacteria metabolism (non-ureolytic pathway). The process involves the carbon cycle through the oxidation of organic salts, particularly heterotrophic sources, such as calcium lactate (CaL), to produce carbonate minerals [18]. The reaction can be described as follows [19]:

CaC_6_H_10_O_6_ + 6O_2_ → CaCO_3_ (solid) + 5CO_2_ + 5H_2_O (6)

Through these processes (ureolytic and non-ureolytic), the precipitates fill the pores of the soil matrix and bind it together, leading to a reduction in permeability [20], as well as an enhancement of compressive strength, shear strength [21], and other mechanical properties of the soil. However, the improvement of soil properties is influenced by the degree of cementation itself, which is determined by the amount and types of precipitated crystals. 

Many researchers have evaluated the factors affecting the degree of cementation, such as the types of bacterial strains, the concentration of bacteria cells, the composition of cementation solution (the type of chemical compounds used) and the concentration of individual chemicals in the solution, environmental conditions, and soil properties [13]. The type and concentration of chemicals used in MICP play a crucial role in controlling the calcium and carbon source [22] as well as contribute to the types and amount of precipitation. The most commonly selected chemicals for MICP are urea, which is hydrolyzed to carbonate ions [23], and calcium chloride, as a source of calcium ions reacting with carbonate ions (Table 1).

However, the morphology (type, shape, and size) of CaCO_3_ crystals can vary depending on the chemical source and can affect the mechanical properties of bio-cemented soil. The types of crystals precipitating during the MICP process, such as calcite, vaterite, or aragonite, have different crystal structures with the same chemical formula [38]. Among these crystals, the most stable form of CaCO_3_ is calcite, which has a simpler rhombohedral structure [39]. Research has been performed on alternative chemical sources that produce more suitable types of precipitation. The study presented by Gorospe et al. [28] showed that using different types of calcium sources produces different characteristics of crystal precipitation. The use of calcium lactate resulted in the largest calcite crystals, followed by calcium acetate, calcium chloride, and then calcium gluconate as the smallest calcite crystals [28]. The study conducted by Fukue et al. [40] revealed that, by increasing the amount of available magnesium ions, dolomite and magnesite crystals are produced, which are considered to be more acid-resistant than calcite. According to the literature, the soil samples treated with a solution containing Mg (Mg/Ca molar ratio = 0.5) were stronger than the samples treated with a solution without magnesium [41], which makes this approach more justified from a technical point of view. In addition, a more accurate recognition of MICP processes in the presence of both metal ions (calcium and magnesium) is supported by the fact that such carbonate precipitation conditions are similar to those in the seas. Magnesium and calcium ions could be found naturally in seawater [42], which may possibly contribute to increasing the efficiency and reducing the cost while stabilizing the coastal area [43]. The pattern of carbonate precipitation at the pore scale is also affected by the concentration of chemicals applied during the treatment stage [22]. Not only do chemicals affect the type and amount of precipitation crystals, but the concentration of bacterial cells also directly affects the kinetics and quality of calcium carbonate precipitation. Specifically, a high rate of carbonate precipitation occurs at high bacterial cell concentrations [44]. A greater concentration of bacterial cells near the particle contacts directly results in increased calcite precipitation in that region [2]. It is also worth mentioning that the growth medium is necessary for bacteria to grow, and its composition depends strictly on the type of bacteria strain. However, the chemicals present in the growth medium should not have a direct impact on the precipitation (except of the number of bacteria cells). The more complex the growth medium, the more nutrients and byproducts may affect the metabolism of the bacteria and the MICP process. As a consequence, the composition of growth medium should be as simple as possible, ensuring, at the same time, the bacteria population development.

Hence, this paper evaluates the chemical composition and concentration of individual components of bio-cementation solution to obtain the optimum combination that produces the highest and most stable amount of carbonates during the MICP process using an environmental strain of *B. subtilis* to support the cementation of fine sand. A new approach in this study was the incorporation of CaL alongside urea as a carbon source, which could potentially reduce the environmental impact of MICP technology. In addition, to the best of the authors’ knowledge, the effect of CaL and Mg ions addition (together) on the efficiency of soil improvement processes has not been tested before. It should be noted that this study used the soil with characteristics that indicate a lower effectiveness of the MICP method. This study was conducted with a focus on a one-cycle application of solutions with varying concentrations of urea, calcium lactate, calcium chloride, magnesium chloride, and bacterial cells expressed as OD. The study setup was designed to determine the effect of (1) the type of dissolved inorganic carbon (DIC) source (urea and calcium lactate), (2) the ratio of Mg_2+_ to Ca^2+^ ion concentrations, and (3) the presence of microorganisms on the mass, nature, and form of the precipitate, as well as the comprehensive strength of the soil after treatment.

## 2. Materials and Methods

### 2.1. Materials

#### 2.1.1. Soil

**Figure 1:**
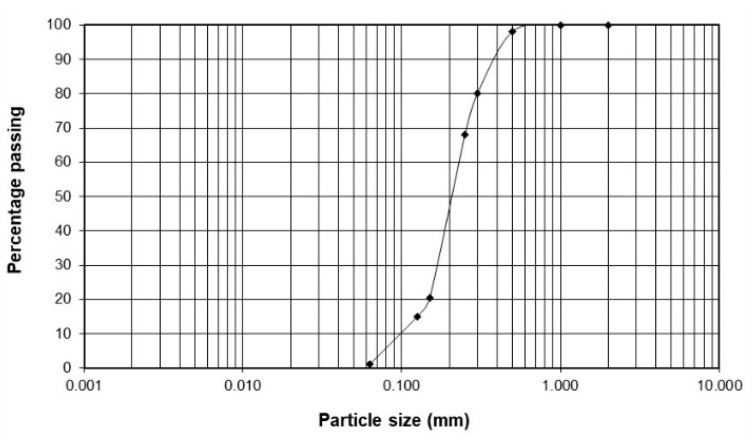
Particle size distribution curve of the tested sand.

The experimental tests were conducted using silica sand obtained from Niemce mine, Lublin voivodeship, Poland. The soil was classified according to the ASTM D 2487–06 standard practice for soil classification as poorly graded sand with grain sizes ranging from 0 to 0.25 mm (Figure 1).

The characteristics of the tested soil are summarized in Table 2. Prior to the test, the sand was sterilized at 105 °C to eliminate the native bacteria naturally present in the soil.

#### 2.1.2. Bacteria Strains

In this study, vegetative cells of *Bacillus subtilis* (obtained from the Institute of Microbiology, Faculty of Biology, University of Warsaw’s own strain collection of environmental bacteria) were used. The bacteria strain was described in detail by Poszytek et al. [45]. The bacterial solutions corresponding to OD_600_ = 1 were prepared using a sterile liquid medium containing final concentrations of 0.5% glucose, 0.25% yeast extract, 0.002 mol/L MgSO_4_, and 0.001 mol/L CaCl_2_. The bacteria were cultured in an oscillating incubator at 30 °C with a vibration rate of 180 rpm for 24 h. The cell concentration was evaluated using a 721 G visible spectrophotometer at a wavelength of 600 nm. Prior to treatment, the liquid bacterial culture had a pH ranging from 5.32 to 5.82 and electrical conductivity in the range of 177.38 to 184.02 µS/cm.

#### 2.1.3. Cementation Solutions

**Figure 2:**
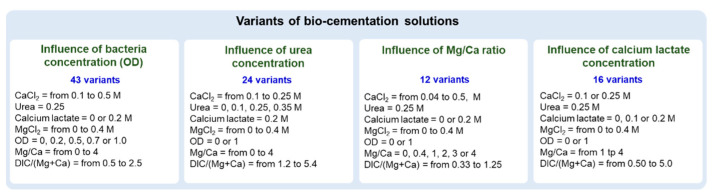
An overview of the bio-cementation solutions used.

The choice of components included in the cementation solutions should primarily depend on the expected metabolic pathway of carbonate precipitation [46]. Therefore, assuming the ureolytic pathway, urea and calcium chloride were analyzed as the main components of the solutions used. Considering the higher strength of precipitates containing magnesium ions (mainly dolomite) compared to calcium carbonates, we also decided to include magnesium chloride at different concentrations and ratios compared to calcium. Additionally, to reduce the usage of urea and the emission of ammonium ions, calcium lactate (CaL) was selected as a source of calcium and organic carbon. The individual chemicals used to prepare the cementation solutions have molar concentrations in the range from 0.1 M to 0.5 M, which, according to [47], may be considered as low and high concentrations, respectively. The compositions and concentrations of the various components of the bio-cementation solutions were chosen to allow deductions to be made about the influence of (1) *B. subtilis* cell concentration (expressed as OD), (2) calcium lactate concentration, (3) urea concentration, and (4) Mg/Ca molar ratio on the efficiency of sand cementation. In total, tests were conducted for 66 different variants, each repeated at least twice. The concentration ranges of individual chemicals used in the current tests are shown in Figure 2, and the compilation of all variants tested is presented in Table S1.

### 2.2. Sample Preparation and Treatment

**Figure 3:**
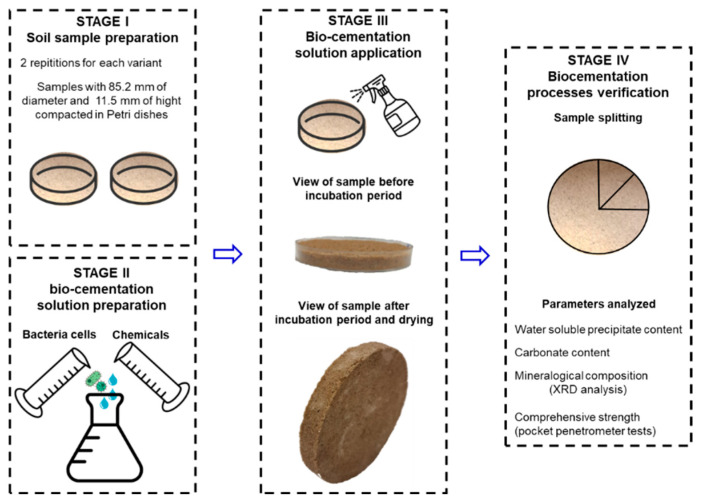
A sequence of steps in soil preparation, treatment, and verification of the effectiveness of bio-cementation.

The dry sand was packed into a Petri dish with an inner diameter of 85.2 mm and a height of 11.5 mm, ensuring that the sample surface was 2 mm below the top of the dish. For each sample, approximately 94 ± 5 g of sand was used, corresponding to a dry density of 1.54 t/m^3^ and a relative density of 61%. Before pouring the bio-cementation solution, the sand was compacted, and the upper surface of the sample was flattened using a wooden cylinder. The specimens were saturated by using a mixture of liquid bacteria culture and cementation solution (referred to as the bio-cementation solution) until an excess of the solution (approximately 3 mm) formed above the sand level. Subsequently, the samples were incubated for 14 days, with all samples being covered during the incubation period to prevent rapid evaporation. Afterwards, the samples were dried in an oven at 105 °C for 24 h to stop microbial activity and remove moisture. The samples were then divided into three parts to verify the effectiveness of the MICP process. The stages of sand sample treatment are schematically shown in Figure 3.

### 2.3. Methods for Verifying the Bio-Cementation Process

#### 2.3.1. Methods for Verifying the Bio-Cementation Process

The water-soluble precipitate and carbonate content (WSC and CC, respectively) were determined using the mass method and washing method [48]. The mass method relies on determining the mass difference in the soil sample before (M_b_) and after being in contact with the reactant (M_a_). To eliminate the water-soluble precipitate, the samples were washed with distilled water on filter paper (size No. 200 sieve) after treatment. Hydrochloric acid solution (1 M) was used as the reactant to determine the carbonate content. All samples were weighed on a laboratory analytical balance (Radwag, Radom, Poland AS 310/C/2) with a precision of ± 0.0001 g. The WSC and CC (expressed as a percentage) were calculated using the formula ((M_a_ − M_b_)/M_b_) × 100%.

#### 2.3.2. Phase Composition Analysis

The phase composition was determined using a Panalytical X’pert PRO MPD X-ray diffractometer equipped with a PW 3020 goniometer, a reflective graphite monochromator, and a Cu copper lamp (CuKα = 1.54178 Å). Measurements were performed in the 1.2–65°2θ range, with a measurement step of 0.02°2θ and duration of 5 s. The obtained XRD spectra were analyzed using the X’Pert Highscore software with the PDF-2 release 2010 database formalized by JCPDS-ICDD. Prior to the analysis, each XRD sample was milled using a milling machine (Planetary Mono Mill Pulverisette 6, Poznan, Poland) at a speed of 400 rpm for 4 min.

#### 2.3.3. Pocket Penetrometer Test

The compressive strength of the soil was measured using a pocket penetrometer to examine the changes in mechanical properties of the soil after treatment with different bio-cementation solutions. The sample was placed on a flat surface, and the compressive strength of soil in kg/cm^2^ was measured using a Durham Geo-Slope Indicator S-170 pocket penetrometer, Szczecin, Poland.

## 3. Results 

### 3.1. Influence of Bacteria Concentration

**Figure 4:**
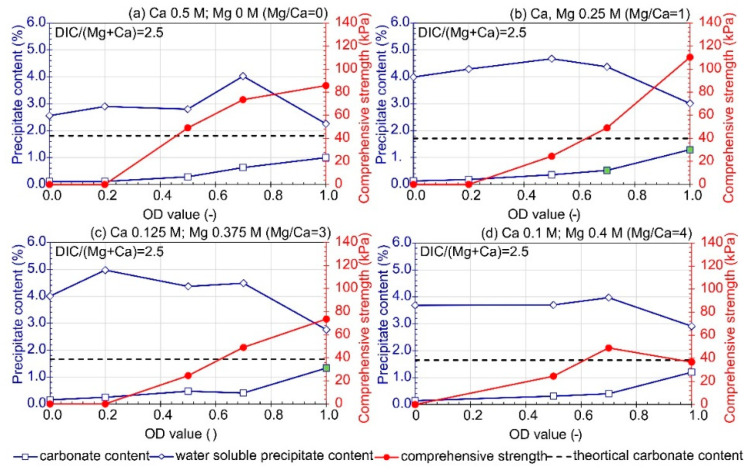
Influence of bacteria concentration (expressed as OD) and Mg/Ca ions ratio on carbonate content, water soluble precipitate content, and comprehensive strength (CaL = 0.2 mol/L, urea = 0.25 mol/L; average value at least *n* = 2). (**a**) Mg/Ca = 0, (**b**) Mg/Ca = 1, (**c**) Mg/Ca = 3, (**d**) Mg/Ca = 4. The green marker indicates a well-cemented sample based on macroscopic evaluation.

**Figure 5:**
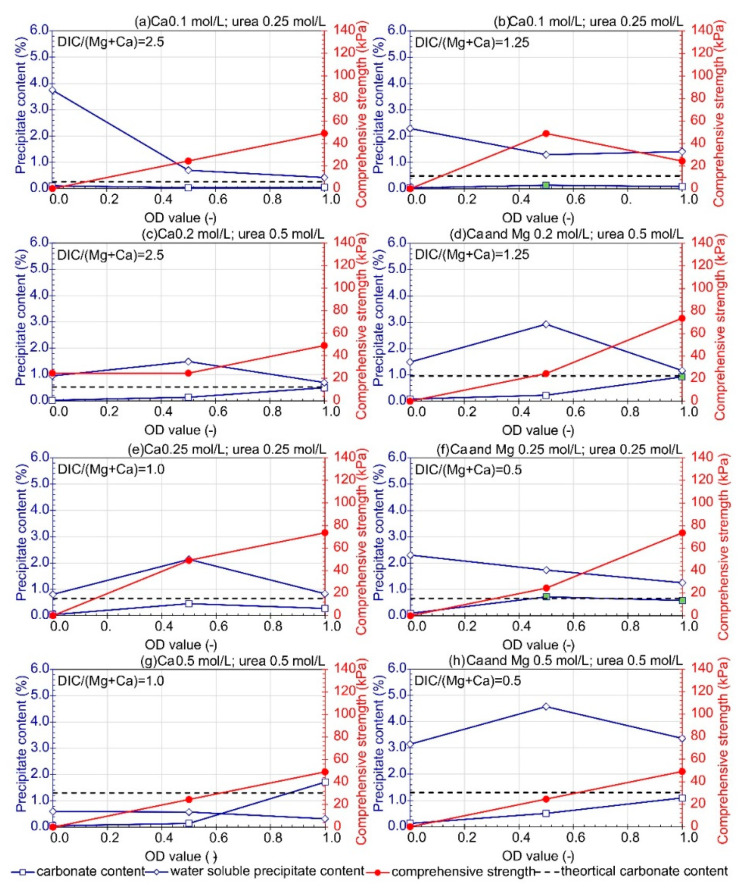
Influence of bacteria concentration on carbonate content, water soluble precipitate content, and comprehensive strength with urea as the source of carbon (average value at least *n* = 2). (**a**) Ca 0.1 M; urea 0.25; DIC/(Mg+Ca) = 2.5, (**b**) Ca 0.1 M; urea 0.25; DIC/(Mg+Ca) = 1.25, (**c**) Ca 0.2 M; urea 0.5; DIC/(Mg+Ca) = 2.5, (**d**) Ca 0.2 M; Mg 0.2 M; urea 0.5; DIC/(Mg+Ca) = 1.25, (**e**) Ca 0.25 M; Mg 0.25 M; urea 0.25; DIC/(Mg+Ca) = 1.0, (**f**) Ca 0.25 M; Mg 0.25 M; urea 0.25; DIC/(Mg+Ca) = 0.5, (**g**) Ca 0.5 M; urea 0.5; DIC/(Mg+Ca) = 1.0, (**h**) Ca 0.5 M; Mg 0.5 M; urea 0.5; DIC/(Mg+Ca) = 0.5. The green marker indicates a well-cemented sample based on macroscopic evaluation.

Soil improvement using the MICP process is associated with the use of native or augmented microorganisms. Specifically, in terms of bioaugmentation, reducing the concentration of bacterial cells in the solution can lead to cost reduction. However, studies have shown a significant increase in precipitation efficiency with higher bacterial concentrations expressed as OD (Figure 4 and Figure 5).

It is important to note that the current studies have not analyzed the biostimulation capability of bioaugmented or native bacteria. In the tests conducted with the presence of CaL (Figure 4), the highest carbonate precipitation and comprehensive strength were observed for the sample with OD equal to 1 (corresponding to a cell concentration of 10^8^ cfu/mL). Simultaneously, these variants showed the lowest content of water-soluble forms. On the other hand, all samples treated with a solution of OD equal to 0 and 0.2, regardless of the concentration of other components, exhibited water absorption properties (sample moisture content higher than 1.1% after 3 days of storage under room conditions; the samples were wet in macroscopic evaluation). Among the variants shown in Figure 4, only three combinations resulted in a well-cemented product (indicated with a green marker on the charts). These samples remained stable after drying at 105 °C, maintaining their disk shape and showing no water absorption from the air. The highest carbonate content was observed for the samples treated with a bio-cementation solution with OD = 1, Mg/Ca ratio = 3 (obtained for CaCl_2_ concentration of 0.125 M and MgCl_2_ concentration of 0.375 M) and containing 0.25 M urea and 0.2 CaL (Figure 4c). However, the highest comprehensive strength measured by the pocket penetrometer was determined for the soil treated with a solution containing Ca and Mg at 0.25 M each and OD equal to 1. In both cases, the sum of added metals was 0.5 M, and the variants differed only in the concentration of individual metals. XRD pattern analysis showed no significant differences in the form of precipitated carbonates. Dolomite, calcite, and aragonite were observed for both variants. Under the adopted test conditions, the proportion of carbonate content (CC) in the total mass of precipitates did not exceed 32.5%. A high contribution of water-soluble forms was observed for all tested variants, which may indicate underutilization of supplied reagents in the processes of urea hydrolysis and CaL decomposition (via bacterial metabolism), as well as carbonate precipitation, even though bacterial cells are present. In this experimental setup, the ratio of dissolved inorganic carbon to the sum of dissolved metals (Ca and Mg) was 2.5, indicating that the process was controlled by the availability of metals. However, for OD equal to 1, the obtained values of CC were close to the theoretical values of carbonate after treatment. The lines representing the theoretical values of CC were calculated assuming complete hydrolysis of urea and decomposition of CaL. The amount of dissolved inorganic carbon (DIC) can be determined from Equations (1)–(3) and (6).

**Figure 6:**
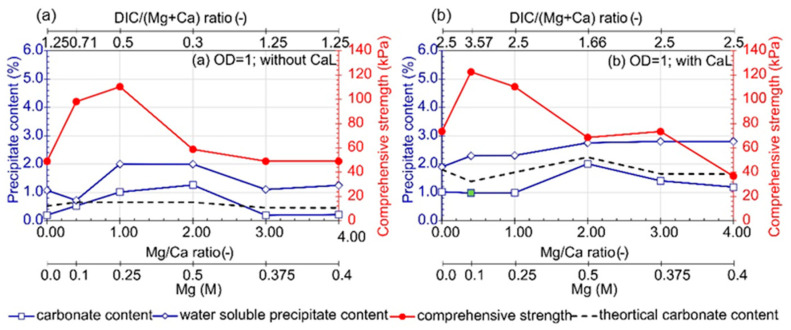
Influence of Mg/Ca ions ratio with (**a**) or without CaL (**b**) presence on carbonate content, water soluble precipitate content, and comprehensive strength (urea concentration 0.25 M, CaL concentration 0 M (**a**) and 0.2 M (**b**); average value, at least *n* = 2). The green marker indicates a well-cemented sample based on macroscopic evaluation.

**Figure 7:**
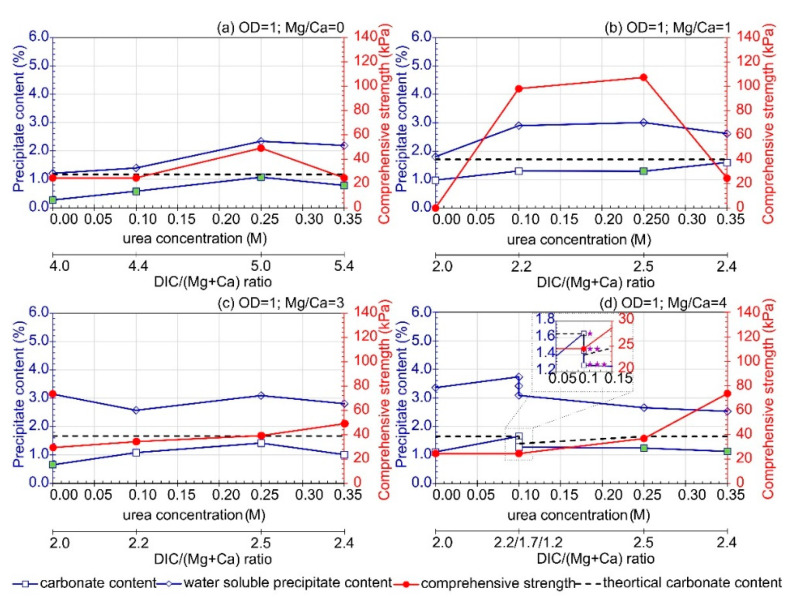
Influence of urea concentration on carbonate content, water soluble precipitate content, and comprehensive strength with Mg/Ca = 0 (**a**), Mg/Ca = 1 (**b**), Mg/Ca = 3 (**c**), and Mg/Ca = 4 (**d**) (average value, at least *n* = 2); purple stars distinguish samples with different concentrations of calcium lactate (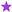
—0.2 M CaL), (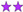
—0.15 M CaL), (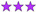
—0.1 M CaL). The green marker indicates a well-cemented sample based on macroscopic evaluation.

**Figure 8:**
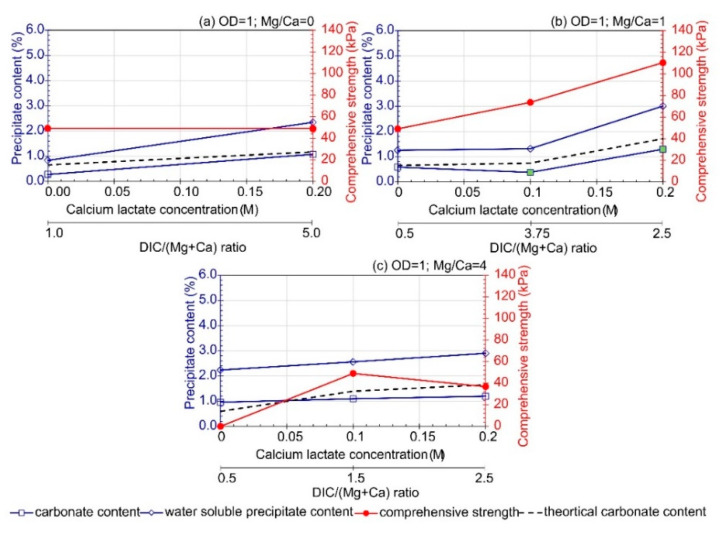
Influence of calcium lactate presence depending on Mg/Ca ions ratio on carbonate (CC), water soluble precipitate content (WC), and comprehensive strength of soil after treatment (average value, at least *n* = 2). The green marker indicates a well-cemented sample based on macroscopic evaluation. (**a**) Mg/Ca = 0, (**b**) Mg/Ca = 1, and (**c**) Mg/Ca = 4.

The theoretical maximum level of carbonate content for the test conditions (marked with a dashed line in the figures) was calculated under simplifications, assuming that, when the amount of dissolved inorganic carbon (DIC) determined the precipitation, calcium ions (Ca^2+^) are consumed first, and magnesium ions (Mg^2+^) are utilized only if carbonate ions (CO_3_^2−^) remain available. Additionally, the ratio of DIC to the sum of available metal ions is presented in Figure 4, Figure 5, Figure 6, Figure 7 and Figure 8, calculated assuming the most favorable conditions, which involve complete urea hydrolysis and CaL decomposition.

Variants with CaL-free solutions were tested with three bacterial cell concentrations: OD equal to 0, 0.5, and 1.0. The variants differed in metal (Ca and Mg) and urea concentration (Figure 5). When comparing the variants with different constituent concentrations (between Figure 5a and Figure 5c, Figure 5b and Figure 5d, Figure 5e and Figure 5g, and Figure 5f and Figure 5h), the reagent concentrations increased twofold while maintaining a constant pairwise ratio of dissolved inorganic carbon (DIC) to the sum of Ca and Mg. It can be observed that higher constituent concentrations increased the precipitation of carbonate forms for an OD of 1, reaching values close to the maximum theoretical carbonate content. This may indicate sufficient microbial activity but inadequate availability of carbon sources necessary for the MICP process (urea and CaL) to occur. However, particularly for the variant with the highest concentrations (0.5 M for each component—Ca, Mg, and urea), the highest proportion of water-soluble forms can be observed in relation to the entire soil sample (amounting to 3.361% for OD = 1). Additionally, for this treatment solution, macroscopic analysis indicated that all samples had a propensity to adsorb water from the environment regardless of bacterial concentration. A possible explanation for this phenomenon could be the crystallization of unconsumed salts. XRD spectra of soil samples with strength values below the limit of quantification (assigned a value of 0 on the graph) revealed the presence of water-soluble precipitates, such as nitrocalcite and nitromagnesite, which absorb moisture from the air.

When analyzing all variants with OD set to 1, it was observed that the highest content of carbonate forms was obtained for the bio-cementation solution containing either 0.5 M or 0.25 M of urea, magnesium, and calcium each (Figure 5f,h). However, after curing, only the sample treated with the 0.25 M solution showed good cementation. This sample had half the amount of water-soluble precipitate compared to the sample treated with a solution with higher component concentrations. The well-cemented samples are indicated by filling in the marker corresponding to that variant in green on the chart (Figure 5).

In terms of the quality of the cemented product formed, the presence of bacteria (expressed as OD) greater than 0.5 resulted in higher stability of the cemented soil. Similar strength values were observed for the soils treated with the following solutions: (1) 0.25 M of urea and Ca, (2) 0.25 M of urea, Ca, and Mg, and (3) 0.5 M of urea and 0.2 M of Ca and Mg. However, these values were lower than in the series of tests with CaL (the best results were obtained for the samples treated with a solution of 0.25 M urea, Ca, and Mg with the addition of 0.2 M CaL), which may indicate that the addition of this compound positively affects the bio-cementation process of fine sand.

The obtained results indicated a wide variability in the efficiency of the MICP process depending on the composition of the bio-cementation solution. Therefore, in the subsequent part of this study, it was decided to evaluate selected aspects in more detail. Additionally, for each variant of solutions containing bacteria, a control test was also conducted without bacteria. For the other variants presented in the following subsections, the test results for samples with OD = 0 are provided only in the Supplementary Materials. 

### 3.2. Influence of Mg/Ca Molar Ratio

The highest carbonate content (CC) values were observed for the samples with an Mg/Ca molar ratio equal to 2 (Figure 6), even though the Mg concentration was 0.5 M.

However, these samples absorbed water from the environment, leading to instability and relatively low comprehensive strength. It should be noted that, for this variant, the total sum of metal ions (Ca and Mg) was the highest, with a value of 0.75 M, while the ratio of dissolved inorganic carbon (DIC) to the mass of metals (Ca and Mg) was the smallest. In this case, for the sample without CaL, the concentration of CO_3_^2−^ was the limiting factor for carbonate precipitation. Despite the excess of metals, a significant amount of carbonates precipitated (markedly above the line representing theoretical values), which may indicate the dissolution of CO_2_ from the atmosphere. For the samples with Mg/Ca molar ratios ranging from 0.4 to 2.0, the amount of precipitated carbonate was governed by the concentration of DIC. In addition to CO_3_^2−^ resulting from urea hydrolysis and CaL metabolism, CO_2_ from the atmosphere may have dissolved and subsequently reacted with available metal ions. This phenomenon could explain the higher measured CC values compared to theoretical values. For the variants without CaL addition, the mass of precipitated carbonate forms does not depend on the magnesium to calcium molar ratio, but rather on the sum of Ca and Mg ions. This is confirmed by the similar CC values observed for the variants with Mg/Ca molar ratios of 0, 0.4, and 4, where the sum of Mg and Ca was 0.2 M (Figure 6a). Additionally, it should be emphasized that all samples were assessed as moderately cemented after curing.

When the cementation solution was enriched with CaL (Figure 6b), the samples with an Mg/Ca molar ratio equal to or greater than 2 became unstable due to water adsorption from the environment. Only the sample with an Mg/Ca molar ratio of 0.4 was classified as well cemented, despite the clearly higher CC observed in the presence of CaL, which was likely due to an additional source of insoluble carbon in the urea. Simultaneously, the content of water-soluble forms also increased. Interestingly, in this series of tests, the samples containing CaL exhibited the highest strength compared to those without CaL. Regardless of the presence of calcium lactate, the most significant soil improvement was observed for Mg/Ca molar ratios of 0.4 and 1, corresponding to a metal sum of 0.35 and 0.5 M, respectively.

It can also be concluded that the lack of bacterial augmentation affected the formation of mainly water-soluble forms. The CC accounted for only 2.6% to 5.8% of the total mass of the precipitate (Figure S1). The presence of bacteria allowed an increase in carbonate content in relation to the total mass of the precipitate, with values ranging from 15% to 42%. In general, an increasing sum of Mg and Ca ion concentrations caused an increase in the proportion of WSC in the total precipitate content. This increase was closely related to the underutilization of the supplied components in the cementation processes that took place.

### 3.3. Influence of Urea Concentration

When analyzing the test results obtained, it can be concluded that, in general, increasing the urea concentration led to an increase in precipitation. However, this trend was observed only up to a certain level of urea concentration, beyond which carbonate precipitation began to slightly decrease (Figure 7).

This decrease may be attributed to a decrease in microbial activity in the presence of urea concentrations above 0.25 M. However, it should be noted that this dependency is also influenced by the availability of magnesium and calcium ions. In the variants without magnesium, all samples after treatment were classified as well-cemented regardless of the urea concentration. However, the comprehensive strength achieved was not satisfactory. This observation suggests that the presence of magnesium has an influence on the precipitation processes taking place. Under the least favorable conditions adopted in the current studies, which consisted of a small population of bacteria involved in urea hydrolysis and CaL decomposition, as well as relatively small weights of added substrates compared to the weight of the treated soil, it is most likely that unused magnesium compounds negatively affect the stability of the precipitated forms. Comparing the variants marked with asterisks (Figure 7d) revealed that, even at a low urea concentration (0.1 M), the presence of CaL at 0.2 M enabled us to obtain very satisfactory results in terms of both the amount of precipitated carbonates and the stability of the formed crystals (sample classified as well-cemented).

Similarly as for carbonate content, an increase in compressive strength can be observed along with an increase in the concentration of urea (Figure 7). It was observed that, for the samples treated with the solution characterized by Mg/Ca molar ratio equal to 0 and 1, an increase in urea concentration above 0.25 M caused a decrease in soil strength. In contrast, a higher proportion of magnesium ions (Mg/Ca equal to 3 and 4) and urea of 0.35 M caused an increase in comprehensive strength, which may indicate a synergistic effect of the two components. However, in terms of comprehensive strength results, the highest values of this parameter were achieved for urea concentration in the range from 0.10 to 0.25 M and Mg/Ca molar ratio equal to 1.

### 3.4. Influence of Calcium Lactate Concentration

The comparison of the effect of calcium lactate (CaL) addition on the precipitate content under conditions without (Figure S3) and with *B. subtilis* bacteria cells (Figure 8) revealed that the contribution of bacteria decreased the content of water-soluble forms to values ranging from 53% to 71% of the total precipitate content.

The most significant increase in the contribution of poorly soluble forms (carbonates) under CaL addition was observed in the variant without magnesium addition, resulting in up to a 4.7-fold increase in CC. However, the effect of CaL addition decreased along with magnesium ion content. It is important to note that the highest content of carbonates (1.29%) was observed for an Mg/Ca molar ratio equal to 1, among the three analyzed values of this ratio.

The increase in CC in samples treated with CaL was attributed to a potential increase in the concentration of dissolved inorganic carbon (DIC), which may have reacted with metal ions (Ca and/or Mg) to form calcium–magnesium carbonate crystals (according to Equation (2)). During bacterial metabolic processes, one mole of CaL can form crystals of CaCO_3_ and five moles of CO_3_^2−^. The implementation of this compound should help improve the utilization efficiency of the metals used (Mg and Ca). However, it should be noted that, with an increase in the content of carbonates, the content of water-soluble forms also increased substantially (WSC). This suggests that the addition of CaL increased the efficiency of the carbonate precipitation reaction, but the concentration of bacteria per gram of soil should be increased to promote more intensive urea hydrolysis and CaL decomposition processes. This conclusion is supported by the fact that increasing the metal concentration from 0.25 M to 0.5 M (Figure 8a,b) resulted not only in an increase in CC but also in WSC, indicating that some of the supplied components were not fully utilized in the precipitation process. Vinjay et al. [49] also observed an increase in CaCO_3_ precipitation with higher concentrations of calcium lactate. However, the use of calcium lactate resulted in the formation of larger vaterite crystals with a spherical shape [38], which could potentially cause inhomogeneous deposition of new crystals during the application of solutions.

The addition of CaL (up to 0.1 M) increased the compressive strength of the samples (Figure 8c) when the Mg/Ca molar ratio was assumed to be 4. However, in the variants with an Mg/Ca molar ratio of 1, the treatment with solution containing CaL resulted in an increase in compressive strength, even at a concentration of 0.2 M. 

### 3.5. XRD Analysis

**Figure 9:**
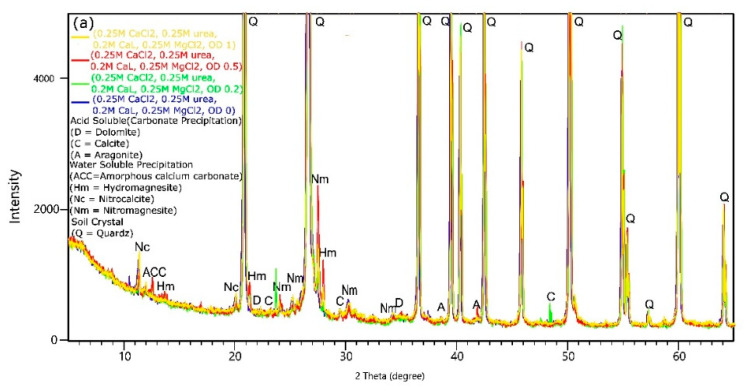

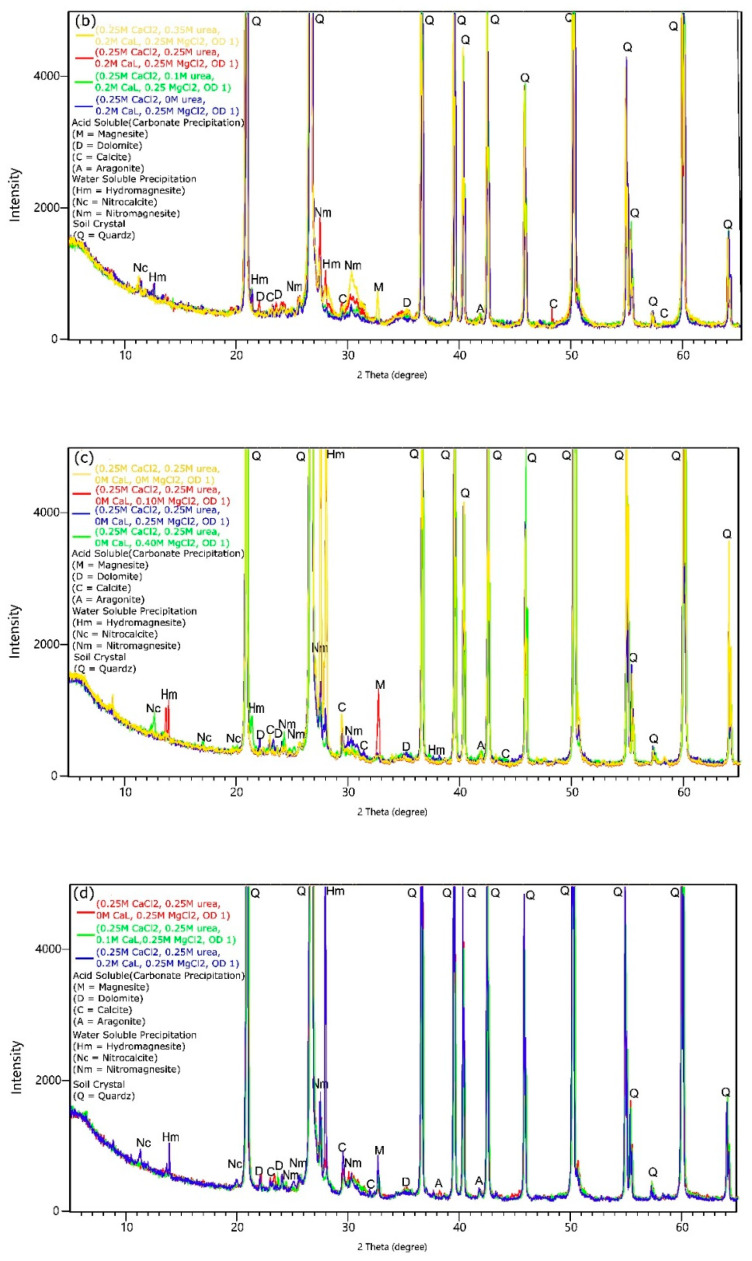
XRD patterns show the influence of (**a**) bacteria concentration (OD), (**b**) urea concentration, (**c**) Mg/Ca molar ratio, and (**d**) calcium lactate concentration on the type of formed crystals.

X-ray Diffraction (XRD) analysis was performed to identify the types of carbonates that precipitated on the sample and provide information about the crystalline phases present in the treated soil. Figure 9a shows a combination of samples with ODs (optical densities) equal to 0, 0.5, and 1.

On the basis of previous results indicating the most effective biostabilization in terms of soil improvement, the combination with CaL (0.2 M) and an Mg/Ca molar ratio of 1 was chosen for a detailed description of the mineral composition of the samples. The analysis revealed that in the sample treated with solutions containing no bacterial cells, only water-soluble precipitates such as nitrocalcite (Ca(NO_3_)_2_) and nitromagnesite (Mg(NO_3_)_2_) were identified. These compounds are colorless salts that absorb moisture from the air [50]. However, new peaks were observed in the XRD pattern of the sample treated with a solution of OD equal to 0.2, corresponding to calcite, and in the sample treated with a solution of OD equal to 0.5, which showed the presence of aragonite (as a form of CaCO_3_) and dolomite. According to Fukue et al. [41], among others, dolomite is a more stable form of carbonate than calcite and its presence may contribute to higher strength parameters of the bio-cemented soil. Furthermore, the XRD pattern of the sample treated with a solution of OD equal to 1 revealed the formation of aragonite, calcite, magnesite, and dolomite, which are classified as acid-resistant minerals [40]. New peaks corresponding to nitrocalcite (highly soluble in water) and amorphous calcium carbonate (ACC) were also observed. ACC is the least stable polymorph of calcium carbonate and usually precipitates at higher rates of hydrolysis [51]. According to Cuthbert et al. [52], higher rates of urea hydrolysis lead to faster nucleation and, consequently, smaller crystal sizes. In the current tests, the concentration of bacteria cells (OD) influenced the types of precipitation. At lower OD (representing a lower concentration of bacteria cells), more water-soluble precipitation was formed, which may be related to the lack of available CO_3_^2−^ ions necessary for carbonate precipitation to occur. Increasing the bacteria concentration led to the formation of minerals classified as acid-resistant. Soon et al. [53] stated that soil cementation increases with higher bacteria cell concentrations since higher cell concentrations can enhance precipitation rates. 

To examine the formed precipitates in relation to urea concentration, XRD analysis was conducted for the samples treated using the bio-cementation solution with OD = 1 and urea concentrations of 0, 0.1, 0.25, and 0.35, visually represented in Figure 9b. In these variants, CaL (calcium lactate) was chosen as an additional source of dissolved inorganic carbon (DIC). When no urea was present, the formation of aragonite and chloromagnesite was observed. The addition of urea at a concentration of 0.1 M resulted in the precipitation of nitromagnesite and dolomite. At a concentration of 0.25 M, aragonite, nitrocalcite, hydromagnesite, calcite, dolomite, and magnesite were formed. Tang et al. [13] observed that higher urea concentrations may lead to faster urea hydrolysis, resulting in increased precipitation. On the other hand, Konstantinou et al. [54] noted that the addition of urea enhances bacterial growth and triggers faster urease production. However, in the current research, the opposite tendency was observed for a urea concentration of 0.35 M. The XRD pattern revealed peaks corresponding to aragonite, nitromagnesite, nitrocalcite, hydromagnesite, calcite, dolomite, and magnesite. The precipitation of water-soluble crystals in this variant increased, which is likely due to an excessive amount of components in the bio-cementation solution in relation to the activity of the microorganisms.

To examine the mineralogical composition of precipitates in terms of the Mg/Ca molar ratio, XRD analysis was performed for four combinations with an OD of 1 and Mg/Ca molar ratios of 0, 1, 0.4, and 2 (Figure 9c). When the Mg/Ca molar ratio was equal to 0, only calcite was detected. However, when the applied solutions were enriched with magnesium ions to achieve an Mg/Ca molar ratio of 1, along with calcite, dolomite and chloromagnesite crystals precipitated. According to Xu et al. [55], the presence of Mg causes changes in the crystal structure and shape of carbonates. Reddy et al. [56] claims that the presence of magnesium ions in concentrations greater than 0.01 M results in the precipitation of calcium carbonate, which eventually recrystallizes into low-magnesium aragonite and calcite with distorted morphology. However, as stated by Fukue et al. [41], a high Mg/Ca molar ratio could contribute to the formation of magnesite and dolomite. A further increase in the magnesium concentration (Mg/Ca = 2) resulted in the precipitation of dolomite, calcite, nitromagnesite, and hydromagnesite. Therefore, it can be concluded that adding Mg ions allowed the induction of precipitation of more stable types of crystals, such as dolomite. However, a too high proportion of magnesium led to the formation of more water-soluble crystals. This phenomenon may be attributed to the inhibitory effect of magnesium on bacterial activity or the underutilization of all the supplied components in the carbonate precipitation reaction.

The analysis of XRD patterns aimed at examining the mineral composition of precipitated carbonates in terms of calcium lactate (CaL) addition revealed that the absence of CaL resulted in the precipitation of various carbonates, including dolomite and nitromagnesite (Figure 9d). The addition of 0.1 M CaL promoted the formation of carbonates such as aragonite, calcite, magnesite, dolomite, and hydromagnesite. According to Wang et al. [57], hydromagnesite (Mg_5_(CO_3_)_4_(OH)_2_·4H_2_O), which consists of five magnesium ions combined with four carbonate ions, is the most stable hydrated magnesium carbonate. Increasing the CaL concentration to 0.2 M resulted in additional precipitation of water-soluble crystals such as nitrocalcite and nitromagnesite. 

## 4. Discussion

The analyses conducted in this research lead us to conclude that the process of soil improvement with the MICP method is influenced by various factors. To test the significance of these correlations, a Pearson correlation matrix was prepared for all variables tested together (Table 3).

The analysis clearly shows that comprehensive strength depended on bacteria concentration (OD), while carbonate content depended on OD, available dissolved inorganic carbon (DIC), calcium lactate (CaL) and magnesium chloride (MgCl_2_) concentrations, the sum of magnesium and calcium, and the Mg/Ca molar ratio. This study also verified that an increase in comprehensive strength was correlated with an increase in carbonate content (CC), indicating that soil improvement was a result of carbonate crystal formation as stated in the research that was conducted by Gowthman et al. [58]

During the conducted studies, the precipitation of water-soluble crystalline forms (WSC) was observed, and their proportion in the sample increased along with CaL and magnesium ion concentrations, as well as the Mg/Ca molar ratio. However, it decreased with increasing bacterial cell concentration. This clearly indicates that inadequate matching of the concentration of the components involved in the process (particularly when concentrations are too high) with the activity of the microorganisms resulted in impaired process efficiency. This conclusion is beneficial from both an economic and environmental perspective, as it promotes efficiency in the use of precipitation precursors during in situ application.

It is worth mentioning that, under all tested conditions, the concentrations of CaCl_2_ and urea did not have a statistically significant effect on comprehensive strength and carbonate content. Therefore, to examine the relationships with a smaller number of variables, the results were grouped according to the assumptions shown in Figure 2.

In a series of tests examining the effect of OD on the occurring processes, similar to the overall results, the concentration of bacterial cells affected carbonate content (R = 0.558) and comprehensive strength (R = 0.817), regardless of whether calcium lactate was present in the solution or not. These observations are consistent with literature reports claiming that soil strength increases along with bacterial concentration. Phang et al. [59] observed that increasing the concentration of bacterial cells from 10^7^ cfu/mL to 10^8^ cfu/mL resulted in up to a 30% increase in the UCS value. This increase may correspond to the efficiency of carbonate precipitation related to the hydrolysis rate [60], which increases along with the number of bacterial cells [42]. In a previous study conducted by Shanoon et al. [61], it was stated that low bacterial cell concentrations did not influence an increase in precipitation efficiency; however, solutions with bacterial concentrations higher than OD = 0.5 showed sufficient urease activity. Murungan et al. [44] demonstrated that the completion of urea hydrolysis required a longer time in cementation solutions inoculated with a low amount of bacterial cells due to the insufficiency of bacterial cells, which reduced the level of urease. Nonetheless, Fukue et al. [62] stated that a high initial ratio of Ca^2+^/OD and a high concentration of calcium ions may inhibit the processes of ureolytic MICP. A high concentration of calcium ions may cause an electrochemical gradient leading to outflow of protons from the cell [63]. Additionally, a higher concentration of Ca ions could possibly form a thick double layer of negatively charged bacteria cell and surrounding Ca^2+^, preventing the intake of nitrogen-containing compounds into the cell and disrupting the proper functioning of the cell [60]. In the case of high concentrations of urea, the effect of enzyme denaturation can be observed. However, as much as 3 moles of urea showed good efficiency in the precipitation of calcium carbonate. However, an excess of urea can lead to the formation of additional toxic ammonium ions, which may inhibit bacteria metabolism and thus the MICP process [64]. In the current research, a decrease in calcium carbonate production and an increase in the formation of water-soluble precipitation were observed for the samples treated with solutions containing fewer bacterial cells. In the current tests, the presence of stable carbonate forms (calcite) was already noted in the samples treated with a solution with an OD of 0.2, which, at higher bacterial concentrations, were enriched with aragonite, dolomite, and magnesite. Additionally, in this series, the amount of precipitated water-soluble forms was affected by magnesium ion concentration (R = 0.688) and Mg/Ca molar ratio (R = 0.512), while CaCl_2_ concentration had a statistically significant effect on CC (R = 0.456). Increasing the concentration of magnesium ions and their ratio to the concentration of calcium had a negative effect on the occurring processes due to the appearance of water-soluble components in this case. A reasonable explanation for this phenomenon could be insufficient bacterial activity, which could also be inhibited by a higher concentration of Mg. However, it should be emphasized that the highest comprehensive strength was obtained for the variant in which the Mg concentration reached 0.25 M and the Mg/Ca molar ratio was 1. These results are consistent with literature, indicating that the addition of Mg in the appropriate amount may positively affect the strength parameters of stabilized soil [28,40]. 

When analyzing the effect of the Mg/Ca molar ratio on soil bio-cementation processes, it was found that this parameter influenced comprehensive strength (R = −0.607). In this series of tests, increasing the proportion of magnesium ions had a lesser effect on improving the strength of the soil after treatment. This may suggest that, despite the most favorable Mg to Ca molar ratio for carbonate precipitation being equal to 5, which is a typical value in seawater [65], in the performed study, increasing the amount of Mg had a lower effectiveness in soil improvement. Moreover, there was a positive correlation between carbonate content (CC) and water-soluble crystalline forms (WSC), magnesium chloride (MgCl_2_), and the sum of Ca^2+^ and Mg^2+^. This supports the previous thesis, as an increased amount of WSC (with increasing MgCl_2_ concentration) deteriorated the strength parameters of the soil. However, from a practical point of view, the presence of Mg ions might be desired, because they possibly contribute to the formation of more stable dolomite [40]. Lv et al. [17] demonstrated that the addition of magnesium ions to the MICP process effectively increased the precipitation and mechanical properties of bio-cemented sand. As mentioned in the literature, this could be because the magnesium ion may also enhance urea activity [66]. From an economical point of view, the use of an additional component applied to the soil (Mg) increases the costs of preparing the bio-cementation solution, but, at the same time, it can potentially increase the efficiency of the stabilization operations carried out. Although magnesium chloride is not considered to be toxic, its application to the soil, especially in excessive amounts, may have negative effects on plants due to the potential increase in salinity and acidification of the soil medium. However, similar side effects can be observed for calcium chloride, which is commonly proposed as a Ca source in MICP processes. The current study showed that, under the adopted test conditions, the previous observations hold true only when the ratio of Mg to Ca ions was less than or equal to 1. Further increases in the Mg/Ca molar ratio slowed down the MICP processes taking place. More specifically, regardless of the presence of CaL, the highest comprehensive strength was observed for the samples with an Mg/Ca molar ratio of 0.4, followed by an Mg/Ca molar ratio of 1. This is in agreement with the results obtained by Lv et al. [17], who observed that a small amount of Mg^2+^ during MICP treatment could improve the mechanical properties of bio-cemented soil.

Urea concentration is one of the most important factors influencing the MICP process, as urease-producing bacteria accelerate urea hydrolysis and, consequently, the production of carbonate ions [67]. On the other hand, considering the regulations of the European Union, the environmental application of urea without a urease inhibitor should be avoided or, at least, limited. Therefore, the main focus was on evaluating the impact of urea concentration in the presence of CaL as an organic compound that can positively influence the environmental aspect of soil bio-cementation.

In the set analyzing the effect of urea on MICP processes, no statistically significant impact of this component was observed on the form of precipitated crystals (water-soluble and carbonates) and comprehensive strength. This could be due to the presence of CaL in the bio-cementation solution, which, upon decomposition, releases more CO_3_^2−^ ions than urea hydrolysis. According to the literature, higher urea concentrations generally result in faster and more extensive carbonate precipitation, leading to stronger bonding within sand grains with higher carbonate precipitation [68]. According to Dhami et al. [69], the concentration of dissolved inorganic carbon (correlated to the concentration of urea and CaL) can enhance the development and metabolic activity of bacteria, promoting the precipitation of calcium carbonate. Interestingly, excessively high urea concentrations may lead to the production of high concentrations of ammonia, which is detrimental to most bacterial cells [70]. Fernández et al. [71] stated that high concentrations of urea can inhibit bacterial metabolism and enzymatic activity. This phenomenon was caused by encapsulation of bacteria cells, which reduced the flow of nutrient and, as a consequence, led to the death of bacteria. However, in the current research, it was observed that the amount of precipitated water-soluble forms depended on the concentration of CaCl_2_ (R = −0.657) and MgCl_2_ (R = 0.727), as well as the Mg/Ca molar ratio (R = 0.727) and the ratio of DIC to total metals (R = −0.633). Consequently, smaller amounts of these forms were formed at higher concentrations of calcium chloride and stoichiometric excess of DIC relative to metals. This confirms, once again, that the formation of water-soluble crystals was related to the presence of unreacted precipitation precursors. Additionally, MgCl_2_ and DIC/(Mg+Ca) influenced the amount of precipitated carbonate forms (with correlations equal to 0.589 and −0.510, respectively). Thus, the results show that the presence of magnesium ions can stimulate carbonate formation, but the mismatch between the load of precipitation precursors (Mg) and the concentration of carbon sources (urea and CaL), as well as the activity of microorganisms in the soil, increased the precipitation of undesirable crystal forms. In the series analyzing the effect of CaL as a source of carbon, only a correlation between this component and the amount of precipitated water-soluble forms (R = 0.762) was observed. Additionally, a correlation between WSC and available DIC was detected in this series (R = 0.762).

Despite the benefits of using the MICP technique to stabilize weak soils, mainly because the method is seen as environmentally friendly, the processes taking place also have the environmental side effect of emitting ammonia and ammonium ions. Although this aspect has not been the subject of this article, nevertheless, it should be remembered that, due to the possibility of soil contamination and eutrophication of surface water, environmental protection methods should be considered. To date, a number of methods have been developed, among which can be included using zeolites characterized by high sorption capacity against ammonium ions [72,73], application of non-ureolytic precipitation pathways [18], e.g., biological oxidation of calcium lactate considered in the current paper or precipitation of ammonium in the form of struvite [74].

## 5. Conclusions

Summarizing the analyses presented in this paper, it can be concluded that both the composition of the bio-cementation solution (the type of chemical components used) and the concentration of the individual chemicals together with the molar ratios between these components play a major role in the effectiveness of the cementing processes occurring in the soil. The conducted tests allow us to draw the following conclusions:

1. Under the adopted test conditions, increasing CaL concentration resulted in the formation of undesirable water-soluble forms that reduced the effectiveness of the method. Additionally, in the presence of CaL, aragonite, calcite, magnesite, dolomite, and hydromagnesite were formed. However, the question of how CaL affects the amount of precipitated carbonates and the improvement of soil strength parameters has not been answered. Water-soluble content also increased with Mg ions concentrations and Mg/Ca molar ratios, and it decreased with bacteria concentration.
2. An increase in the Mg/Ca molar ratio decreased the efficiency of soil bio-stabilization. However, the presence of Mg ions in lower concentrations stimulated the formation of more stable crystals of dolomite and the improvement of soil. The optimum combination of bio-cementation solution containing *B. subtilis* bacteria strain, assuming a single application treatment, based on the comprehensive strength of cemented sand, is as follows: OD equal to 1, urea concentration of 0.25 M, CaL concentration of 0.1–0.2 M, and the Mg/Ca molar ratio between 0.4 and 1. The highest comprehensive strength was achieved with Mg^2+^ and Ca^2+^ concentrations equal to 0.1 M and 0.25 M, respectively. This composition of bio-cementation solution can be recommended for in situ application; however, the efficiency of the MICP processes may depend on the type of treated soil.
3. The comprehensive strength and carbonate content depended on bacteria cell concentrations. Moreover, the correlation between carbonate content and bacteria concentration, available dissolved inorganic carbon, calcium lactate and magnesium chloride concentration, as well as the sum and molar ratio of Mg and Ca ions, was revealed. Additionally, an increase in comprehensive strength was related to an increase in carbonate content.
4. The results presented in the article, however, do not answer several important questions. Therefore, it would be reasonable to conduct numerical analysis of the precipitation processes to determine the relationships between parameter concentrations and WSC, CC, and comprehensive strength. Additionally, for environmental reasons, further studies are needed to eliminate urea or at least significantly increase the contribution of the non-ureolytic carbonate precipitation pathway in the application of the MICP method under in situ conditions.

## Figures and Tables

**Table 1:** Summary of bacterial strains and characteristics of solutions used in MICP—literature review.

Bacteria Strain	Growth Medium	DIC Source (Concentration)	Metal (Ca^2+^) Source (Concentration)	Additional Components (Concentration)	Refs.
*B. pasteurii* (ATCC 6453)	urea growth medium (3 g/L bacteria nutrient broth, 20 g/L urea, 10 g/L NH_4_Cl, 2.12 g/L NaHCO_3_, 25.5 mM CaCl_2_), pH 7.5	urea (20 g/L or 0.33 M)	CaCl_2_ (140 g/L or 1.26 M)	-	[24]
*S. pasteurii* (DSMZ 33)	growth medium (20 g/L YE, 10 g/L NH_4_Cl, pH 9.0)	urea (1.1 M)	CaCl_2_ (1.1 M)	fixation solution: CaCl_2_ (0.05 M)	[25]
*S. pasteurii* (DSM 33)	nutrient broth containing (20 g/L), yeast extract, NH_4_Cl (10 g/L), NiCl_2_ (10µM)	urea (1 mol/L)	CaCl_2_ (1.1 mol/L)	-	[26]
*S. pasteurii* (DSMZ 33)	-	urea (1.0 M)	CaCl_2_ (1.0 M)	fixation solution: CaCl_2_ (0.05 M); deionized water, fresh surface water, saline water (9 g/L NaCl)	[27]
*S. pasteurii* (NO-A10)	growth medium in EDC solution (10 g/L), culture medium, 5% NaCl solution	urea (1.0 M)	MgCl_2_ (0.5 M), CaCl_2_ (0.5 M or 1.0 M)	buffer solution (0.01 M NH_4_OH+NH_4_Cl)	[28]
*S. pasteurii* (ATCC 11859)	NH_4_-YE medium (20 g YE, 10 g (NH_4_)_2_SO_4_, 20 g of agar in 0.13 M Tris buffer, pH = 9.0), OD = 0.8–1.2	-	equimolar urea- CaCl_2_ (0.1, 0.25, 0.5, 1.0 M)	nutrient broth (3 g/L), NH_4_Cl 10 g/L, NaHCO_3_ (2.12 g/L)	[20]
*Bacillus* sp.	-	urea (1.0 M)	CaCl_2_ (0.45 M)	-	[29]
*S. pasteurii* (ATCC 6452)	NH_4_-YE medium (20 g/L YE, 10 g/L (NH_4_)_2_SO_4_, 20 g/L agar in 0.13 M Tris buffer, pH = 9.0)	urea (1.0 M)	CaCl_2_ (1.0 M)	nutrient broth (3 g/L)	[30]
*S. pasteurii* (ATCC 11859)	(1) 0.5 M urea, 0.187 M NH_4_Cl, 3 g/L nutrient broth, (2) 0.1 M CaCl_2_, 0.187 M NH_4_Cl, 3 g/L nutrient broth (increased salinity to enhance the adsorption of microorganisms on sand grains)	urea (0.25 M)	CaCl_2_ (0.25 M)	-	[31]
*S. pasteurii*	NH4Cl (0.006 M and 0.012 M, C2H3NaO2(0.0425 M and 0.085 M, and YE (0.1 and 0.2 g/L)	urea (0.25 M and 0.5 M)	CaCl_2_ (0.125 M and 0.25 M)	C_2_H_3_NaO_2_ (-)	[32]
*S. pasteurii*	growth medium (in 0.13 mol/L tris buffer, 10 g/L (NH4)2SO4, 20 g/L YE)	urea (0.333 M)	CaCl_2_ (0.1 M)	NH_4_Cl (0.374 M)	[33]
*S. pasteurii* (ATCC 1859)	YE (20 g), (NH_4_)_2_SO_4_ (10 g), 0.13 M tris buffer (pH = 9.0) solution	urea (0.3 M)	CaCl_2_ (0.3 M)	-	[34]
*S. pasteurii* (DSMZ 33) + native urease-producing bacteria	ureolytic broth (1 M urea, without (NH_4_)_2_SO_4_)	urea (1.0 M)	CaCl_2_ (1.0 M)	C_2_H_3_NaO_2_ (13.8 g/L)	[35]
*B. sphaericus*	25 g/L nutrient broth	urea (0.5 M)	CaCl_2_ (0.5 M)	NH_4_Cl (10 g/L) and NaHCO_3_ were (2.12 g/L)	[36]
*B. subtilis*	peptone (5 g), NaCL (5 g), YE (4 g), beef extract (1 g), urea (50 mL)	urea (30.03 g/L or 0.5 M)	CaCl_2_ (27.75 g/L or 0.25 M)	NH_4_Cl (10 g), NaHCO_3_ (2.12 g)	[37]
*S. pasteurii* (ATCC 11859)	NH_4_-YE medium (20 g/L YE, 10 g/L (NH_4_)_2_SO_4_, 15.73 g/L Tris base)	urea (0.5 M)	C_4_H_6_CaO_4_, Ca(NO_3_)_2_, and CaCl_2_ (0.5 M).	MgCl_2_ (0, 0.05, 0.1, 0.25, 0.5 M).	[17]

**Table 2:** Characteristics of the tested soil.

Classification		Results
Specific density	t/m^3^	2.593
Dry density min	t/m^3^	1.326
Dry density max	t/m^3^	1.714
pH	-	8.18
D10	mm	0.09
D30	mm	0.17
D60	mm	0.23
Cu	-	2.6
Cc	-	2.4

**Table 3:** Correlation matrix (Pearson) for the tested variables. Values in bold indicate a significance level different from 0 at alpha = 0.05.

Variables	Comprehensive Strength (kPa)	WSC (%)	CC (%)
CaCl_2_ (M)	0.078	−0.063	−0.044
Urea (M)	−0.032	−0.199	−0.210
CaL (M)	0.038	**0.592**	**0.411**
MgCl_2_ (M)	−0.113	**0.589**	**0.442**
OD (-)	**0.606**	**−0.223**	**0.678**
Mg/Ca	−0.144	**0.431**	**0.355**
Σ(Ca, Mg) (M)	−0.051	**0.519**	**0.391**
DIC (M)	0.033	**0.598**	**0.395**
DIC/(Ca+Mg)	0.044	0.194	0.115
Comprehensive strength (kPa)	**1**	−0.180	**0.363**
WSC (%)	−0.180	**1**	0.154
CC (%)	**0.363**	0.154	**1**

## Data Availability

Data are contained within the article and Supplementary Material.

## Supplementary Materials

The following supporting information can be downloaded at: https://www.mdpi.com/article/10.3390/ma16175767/s1, Table S1. Compilation of CaCl2, MgCl2, urea, and calcium lactate concentration in bio-cementation solutions used in individual variants (for each variant, the sample with OD = 0 was additionally tested); Figure S1: Effect of Mg/Ca molar ratio on CC and WSC in samples without bacterial cells (OD = 0) without the presence (a) and in the presence of CaL (b); Figure S2: Effect of urea concentration ratio on CC and WSC in samples without bacterial cells (OD = 0) at Mg/Ca ratios of 0 (a), 1 (b), 3 (c), and 4 (d). The concentration of CaL was equal to 0.2 M; Figure S3: Effect of CaL presence on CC and WSC in samples without bacterial cells (OD = 0).
