# Supplementary material for: Assessment of the Composition Effect of a Bio-Cementation Solution on the Efficiency of Microbially Induced Calcite Precipitation Processes in Loose Sandy Soil
Source: Materials (Basel). 2023 Aug 23;16(17):5767. doi: 10.3390/ma16175767 (PMC10488806; doi:10.3390/ma16175767)
Supplement: Supplementary file 1 [file materials-16-05767-s001.zip › materials-2536298-supplementary.pdf]

materials

MDPI

Supplementary Materials

Assessment of the Composition Effect of a Bio-Cementation Solution on the Efficiency of Microbially Induced Calcite Precipitation Processes in Loose Sandy Soil

Joanna Fronczyk [1,*], Nadella Marchelina [2], Adam Pyzik [2] and Małgorzata Franus [2]

[1] Institute of Civil Engineering, Warsaw University of Life Sciences—SGGW, 166 Nowoursynowska Str., 02-787 Warsaw, Poland
[2] Faculty of Civil Engineering and Architecture, Lublin University of Technology, 40 Nadbystrzycka Str., 20-618 Lublin, Poland; d570@pollub.pl (N.M.); a.pyzik@pollub.pl (A.P.); m.franus@pollub.pl (M.F.)
* Correspondence: joanna_fronczyk@sggw.edu.pl; Tel.: +48-22-59-35-207

Table S1. Compilation of CaCl2, MgCl2, urea and calcium lactate concentration in bio-cementation solutions used in individual variants (for each variant, the sample with OD=0 was additionally tested).

$CaCl_2$	Urea	CaL	$MgCl_2$	OD
0.5	0.25	0.2	0	0.0
0.5	0.25	0.2	0	0.2
0.5	0.25	0.2	0	0.5
0.5	0.25	0.2	0	0.7
0.5	0.25	0.2	0	1.0
0.25	0.25	0.2	0.25	0.0
0.25	0.25	0.2	0.25	0.2
0.25	0.25	0.2	0.25	0.5
0.25	0.25	0.2	0.25	0.7
0.25	0.25	0.2	0.25	1.0
0.125	0.25	0.2	0.375	0.0
0.125	0.25	0.2	0.375	0.2
0.125	0.25	0.2	0.375	0.5
0.125	0.25	0.2	0.375	0.7
0.125	0.25	0.2	0.375	1.0
0.1	0.25	0.2	0.4	0
0.10	0.25	0.20	0.40	0.50
0.10	0.25	0.20	0.40	0.70
0.10	0.25	0.20	0.40	1.00
0.1	0.25	0	0	0
0.1	0.25	0	0	0.5
0.1	0.25	0	0	1
0.1	0.25	0	0.1	0
0.1	0.25	0	0.1	0.5
0.1	0.25	0	0.1	1
0.25	0.25	0	0	0
0.25	0.25	0	0	0.5
0.25	0.25	0	0	1
0.25	0.25	0	0.25	0
0.25	0.25	0	0.25	0.5
0.25	0.25	0	0.25	1
0.5	0.5	0	0	0
0.5	0.5	0	0	0.5
0.5	0.5	0	0	1
0.5	0.5	0	0.5	0
0.5	0.5	0	0.5	0.5
0.5	0.5	0	0.5	1
0.2	0.25	0	0	1

0.25	0.25	0	0.1	1
0.25	0.25	0	0.25	1
0.25	0.25	0	0.5	1
0.05	0.25	0	0.15	1
0.04	0.25	0	0.16	1
0.5	0.25	0.2	0	1
0.25	0.25	0.2	0.1	1
0.25	0.25	0.2	0.25	1
0.25	0.25	0.2	0.5	1
0.125	0.25	0.2	0.375	1
0.1	0.25	0.2	0.4	1
0.25	0	0.2	0	1
0.25	0.1	0.2	0	1
0.25	0.25	0.2	0	1
0.25	0.35	0.2	0	1
0.25	0	0.2	0.25	1
0.25	0.1	0.2	0.25	1
0.25	0.25	0.2	0.25	1
0.25	0.35	0.2	0.25	1
0.125	0	0.2	0.375	1
0.125	0.1	0.2	0.375	1
0.125	0.25	0.2	0.375	1
0.125	0.35	0.2	0.375	1
0.1	0	0.2	0.4	1
0.1	0.1	0.2	0.4	1
0.1	0.1	0.15	0.4	1
0.1	0.1	0.1	0.4	1
0.1	0.25	0.2	0.4	1
0.1	0.35	0.2	0.4	1
0.25	0.25	0	0	1
0.25	0.25	0.2	0	1
0.25	0.25	0	0.25	1
0.1	0.25	0.1	0.1	1
0.25	0.25	0.2	0.25	1
0.1	0.25	0	0.4	1
0.1	0.25	0.1	0.4	1
0.1	0.25	0.2	0.4	1

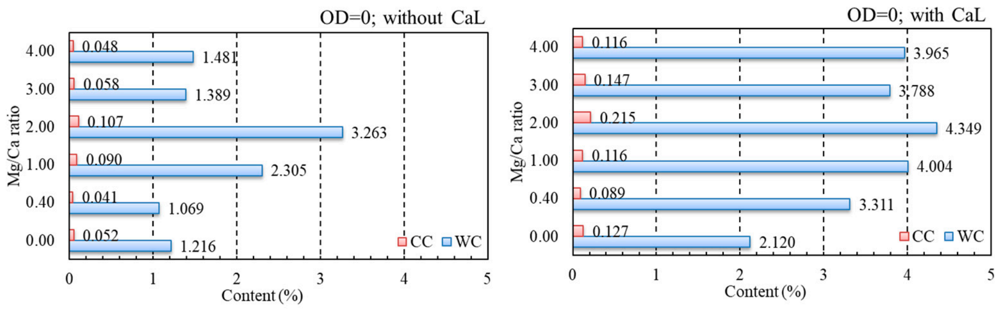

Figure S1. Effect of Mg/Ca molar ratio on CC and WSC in samples without bacterial cells (OD=0) without the presence (**a**) and in the presence of CaL (**b**).

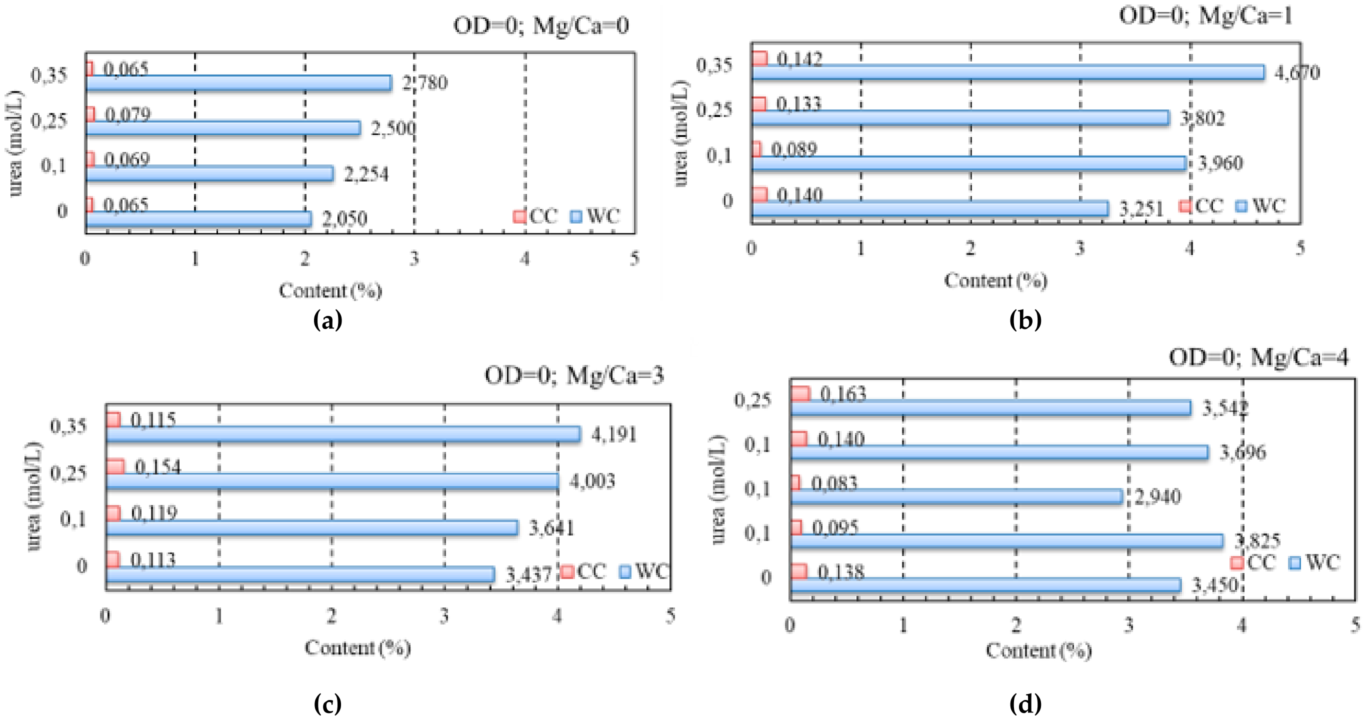

Figure S2. Effect of urea concentration ratio on CC and WSC in samples without bacterial cells (OD=0) at Mg/Ca ratios of 0 (**a**), 1 (**b**), 3 (**c**) and 4 (**d**). The concentration of CaL was equal to 0.2 M.

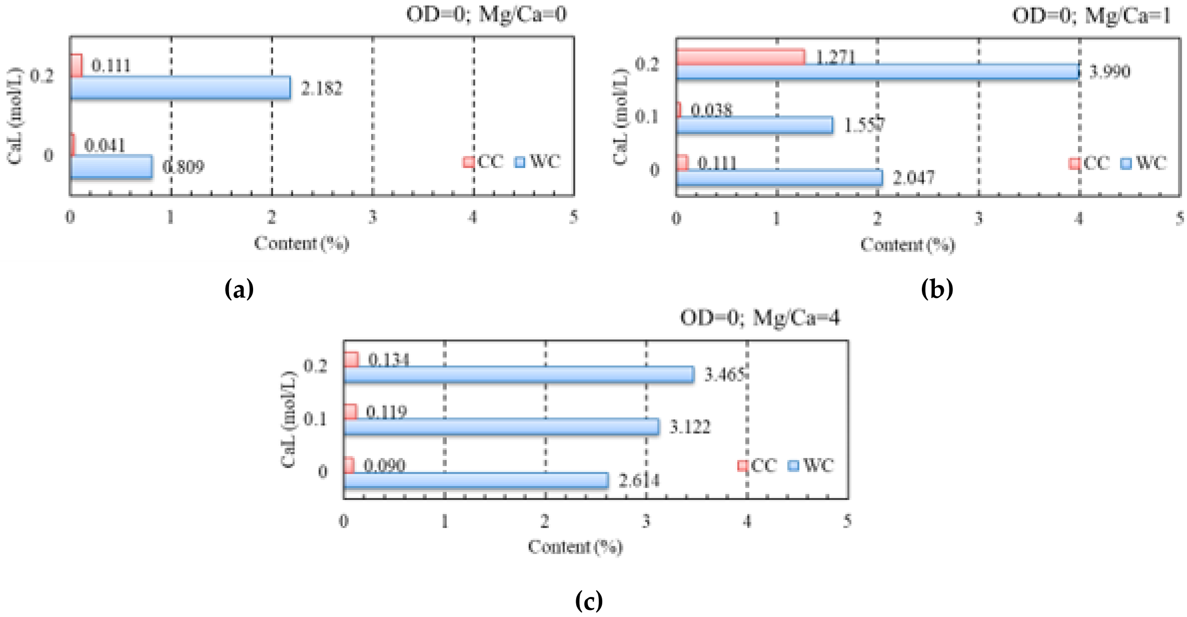

Figure S3. Effect of CaL presence on CC and WSC in samples without bacterial cells (OD=0), at Mg/Ca ratios of 0 (**a**), 1 (**b**), 4 (**c**)
